# 4-(5-Chloro-2-hydroxy­benzyl­idene­amino)-*N*-(4,6-dimethyl­pyrimidin-2-yl)benzene­sulfonamide

**DOI:** 10.1107/S1600536808005606

**Published:** 2008-03-05

**Authors:** Zahid H. Chohan, M. Nawaz Tahir, Hazoor A. Shad, Islam Ullah Khan

**Affiliations:** a Department of Chemistry, Bahauddin Zakariya University, Multan 60800, Pakistan; b Department of Physics, University of Sargodha, Sargodha, Pakistan; c Department of Chemistry, Government College University, Lahore, Pakistan

## Abstract

The title compound, C_19_H_17_ClN_4_O_3_S, is a Schiff base compound of 5-chloro­salicylaldehyde and sulfamethazine [4-amino-*N*-(4,6-dimethyl-2-pyrimidin­yl)benzene­sulfonamide]. The geometry around the S atom is distorted tetra­hedral, comprising two O atoms of the sulfonyl group, a C atom of a benzene ring and the amino N atom. The title compound has an intra­molecular O—H⋯N hydrogen bond and two inter­molecular C—H⋯O and N—H⋯O hydrogen bonds, which link neighbouring mol­ecules into 10-membered rings. As a result of an unavoidable conformational arrangement, a slightly short intra­molecular contact of distance 2.59 Å exists between an O atom of the sulfonyl group and an H atom of the sulfamethazine benzene ring.

## Related literature

For related literature, see: Basak *et al.* (1983 ▶); Chohan & Shad (2007 ▶); Yang (2006 ▶); Shad *et al.* (2008 ▶); Zareef *et al.* (2007 ▶).
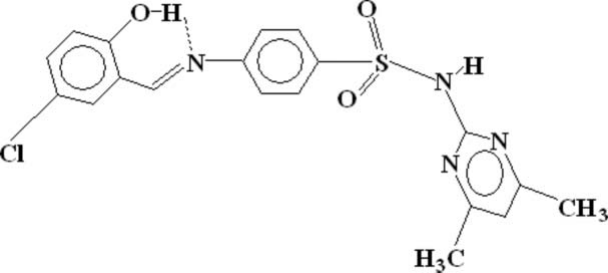


## Experimental

### 

#### Crystal data

C_19_H_17_ClN_4_O_3_S
*M* *_r_* = 416.88
Orthorhombic, 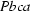

*a* = 11.7332 (7) Å
*b* = 13.8506 (6) Å
*c* = 23.6635 (14) Å
*V* = 3845.6 (4) Å^3^
*Z* = 8
Mo *K*α radiation radiation
μ = 0.34 mm^−1^
*T* = 296 (2) K
0.22 × 0.18 × 0.14 mm

#### Data collection

Bruker Kappa APEXII CCD diffractometer
Absorption correction: multi-scan (*SADABS*; Bruker, 2005 ▶) *T*_min_ = 0.940, *T*_max_ = 0.958
21114 measured reflections
4787 independent reflections
2797 reflections with *I* > 2σ(*I*)
*R*_int_ = 0.043

#### Refinement

*R*[*F* ^2^ > 2σ(*F* ^2^)] = 0.049
*wR*(*F* ^2^) = 0.148
*S* = 1.05
2797 reflections
259 parameters
H atoms treated by a mixture of independent and constrained refinement
Δρ_max_ = 0.32 e Å^−3^
Δρ_min_ = −0.28 e Å^−3^

### 

Data collection: *APEX2* (Bruker, 2007 ▶); cell refinement: *APEX2*; data reduction: *SAINT* (Bruker, 2007 ▶); program(s) used to solve structure: *SHELXS97* (Sheldrick, 2008 ▶); program(s) used to refine structure: *SHELXL97* (Sheldrick, 2008 ▶); molecular graphics: *ORTEP-3 for Windows* (Farrugia, 1997 ▶) and *PLATON* (Spek, 2003 ▶); software used to prepare material for publication: *WinGX* (Farrugia, 1999 ▶) and *PLATON*.

## Supplementary Material

Crystal structure: contains datablocks global, I. DOI: 10.1107/S1600536808005606/fj2100sup1.cif

Structure factors: contains datablocks I. DOI: 10.1107/S1600536808005606/fj2100Isup2.hkl

Additional supplementary materials:  crystallographic information; 3D view; checkCIF report

## Figures and Tables

**Table 1:** Hydrogen-bond geometry (Å, °)

*D*—H⋯*A*	*D*—H	H⋯*A*	*D*⋯*A*	*D*—H⋯*A*
O1—H1⋯N1	0.73 (5)	1.90 (4)	2.534 (3)	146 (5)
N2—H2⋯O1^i^	0.71 (3)	2.22 (3)	2.886 (3)	156 (3)
C9—H9⋯O2^ii^	0.93	2.48	3.398 (4)	171

Symmetry codes: (i) 
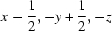
; (ii) 
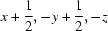
.

**Fig. 1.**
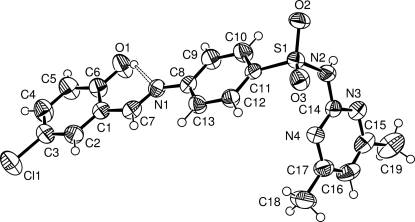
ORTEP-3 for Windows (Farrugia, 1997) drawing of the title compound, C19H17Cl1N4O3S, with the atom numbering scheme. The thermal ellipsoids are drawn at the 50% probability level. H-atoms are shown by small circles of arbitrary radii. The intramolecular H-bonding is shown by dashed lines.

**Fig. 2.**
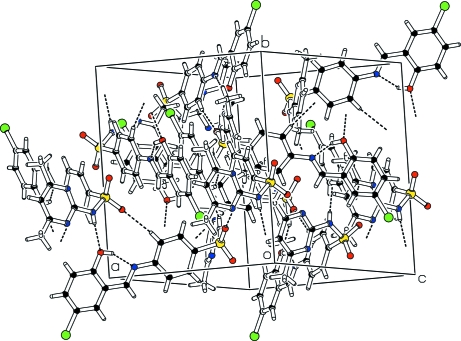
The unit cell packing of (I) (Spek, 2003), showing the intermolecular hydrogen bonding completing a ten-membered ring.

## supplementary crystallographic information

### Comment

Sulfonamides have widely been recognized for their wide variety of pharmacological activities such as antibacterial, antitumor, anti-carbonic anhydrase, diuretic, hypoglycaemic, antithyroid and protease inhibitory activity. Sulfonamides have also been used clinically as antimalarial agents (Zareef *et al.*, 2007), particularly sulfadiazine and sulfadoxine. Due to their significant pharmacology applications and widespread use in medicine, these compounds have also gained attention in bio-inorganic and metal-based (Chohan *et al.*, 2007) drug chemistry. In continuation to the preparation of schiff base ligands with various sulfa drugs and halogen substituated aldehydes (Shad *et al.*, 2008), we report herein the structure of the title compound.

The title compound (I), is a Schiff base ligand containing sulfamethazine and 5-chlorosalicylaldehyde. The crystal structure (Basak *et al.*, 1983) of (II) sulfamethazine:[4-amino-*N*-(4,6-dimethyl-2-pyrimidinyl)benzenesulfonamide] have been reported. The search in Cambridge Crystallographic Data Center showed that no crystal structure of 5-chlorosalicylaldehyde as an individual moiety is reported. Few structures containing the later have been reported such as 5-chlorosalicylaldehyde salicylhydrazone (Yang, 2006). The mutual effect of sulfamethazine and the 5-chlorosalicylaldehyde in solid form is observed by enlarging the bond lengths N1—C8 [1.414 (3) Å], S1—C11 [1.759 (3) Å] compared to 1.367 (3)° and 1.746 (3) Å respectively as reported in (II). The bond distances in the 4,6-dimethyl-2-pyrimidinyl moiety of sulfamethazine remained same whithin experimental errors. The observed bond angles C11—S1—N2 and S1—N2—C14 have values of 106.75 (13)°, 126.2 (2)° in comparison to 108.2 (1)° and 128.0 (2)° respectively. The torsion angle C11—S1—N2—C14: -64.3 (3)° in the title compound compared to 83.0 (3)° shows that the 4,6-dimethyl-2-pyrimidinyl moiety becomes almost in *trans* position. The observed change in 5-chlorosalicylaldehyde moiety, is mainly of bond distances C6—O1 [1.328 (4) Å] and Cl1—C5 [1.736 (3) Å], whereas these values are 1.347 (5) Å and 1.746 (4) Å as reported in 5-chlorosalicylaldehyde salicylhydrazone. The coordinates of H-atoms attached to O1 and N2 were refined. The rings A(C14/N3/C15/C16/C17/N4), B(C1/C2/C3/C4/C5/C6) and C(C8/C9/C10/C11/C12/C13), make dihedral angles A/B[78.95 (8)°], A/C[83.50 (8)°] and B/C[4.57 (19)°] respectively. The sulfonyl group (O2/S1/O3) have a dihedral angle of 56.36 (11)° with ring (B) and with ring (A), it is 54.25 (14)°. An intramolecular H-bond of O—H···N type complete six-membered ring, as shown in Fig 1. The two intermolecular H-bonds of C—H···O and N—H···O form a ten membered ring by sharing a six membered ring due to intramolecular H-bond as in Fig 2. The detail of H-bonds is given in Table 1.

### Experimental

Sulfamethazine (0.5566 g, 2 mmol) in ethanol (15 ml) was reacted with ethanolic (10 ml) solution of 5-chlorosalicylaldehyde (0.3131 g, 2 mmol). The mixture was refluxed for 3 h. The colour of the solution gradually changed from colourless to orange-red. The solution was then cooled to room temperature, filtered and volume reduced to about one-third on rotary evaporator. After 10 days crystals of the title compound were obtained.

### Refinement

The coordinates of the hydroxy and amide H-atoms were freely refined. Other H-atoms were positioned geometrically, with C—H = 0.93 and 0.96 Å for aromatic and methyl carbons respectively. The *U*_iso_(H) = *xU*_eq_(C, N, O), where *x* = 1.5 for methyl H, and *x* = 1.2 for all other H atoms.

### Crystal data

**Table:**

C_19_H_17_ClN_4_O_3_S	*D*_x_ = 1.440 Mg m^−^^3^
*M**_r_* = 416.88	Melting point: 497 K
Orthorhombic, *P**b**c**a*	Mo *K*α radiation radiation λ = 0.71073 Å
Hall symbol: -P 2ac 2ab	Cell parameters from 2797 reflections
*a* = 11.7332 (7) Å	θ = 1.7–28.3º
*b* = 13.8506 (6) Å	µ = 0.34 mm^−^^1^
*c* = 23.6635 (14) Å	*T* = 296 (2) K
*V* = 3845.6 (4) Å^3^	Prismatic, red
*Z* = 8	0.22 × 0.18 × 0.14 mm
*F*_000_ = 1728	

### Data collection

**Table:**

Bruker Kappa APEXII CCD diffractometer	4787 independent reflections
Radiation source: fine-focus sealed tube	2797 reflections with *I* > 2σ(*I*)
Monochromator: graphite	*R*_int_ = 0.043
Detector resolution: 7.40 pixels mm^-1^	θ_max_ = 28.3º
*T* = 296(2) K	θ_min_ = 1.7º
ω scans	*h* = −15→15
Absorption correction: multi-scan(SADABS; Bruker, 2005)	*k* = −17→18
*T*_min_ = 0.940, *T*_max_ = 0.958	*l* = −31→30
21114 measured reflections	

### Refinement

**Table:**

Refinement on *F*^2^	Secondary atom site location: difference Fourier map
Least-squares matrix: full	Hydrogen site location: inferred from neighbouring sites
*R*[*F*^2^ > 2σ(*F*^2^)] = 0.049	H atoms treated by a mixture of independent and constrained refinement
*wR*(*F*^2^) = 0.148	*w* = 1/[σ^2^(*F*_o_^2^) + (0.0488*P*)^2^ + 2.8076*P*] where *P* = (*F*_o_^2^ + 2*F*_c_^2^)/3
*S* = 1.05	(Δ/σ)_max_ < 0.001
2797 reflections	Δρ_max_ = 0.32 e Å^−^^3^
259 parameters	Δρ_min_ = −0.28 e Å^−^^3^
Primary atom site location: structure-invariant direct methods	Extinction correction: none

### Special details

**Table:**

Geometry. All e.s.d.'s (except the e.s.d. in the dihedral angle between two l.s. planes) are estimated using the full covariance matrix. The cell e.s.d.'s are taken into account individually in the estimation of e.s.d.'s in distances, angles and torsion angles; correlations between e.s.d.'s in cell parameters are only used when they are defined by crystal symmetry. An approximate (isotropic) treatment of cell e.s.d.'s is used for estimating e.s.d.'s involving l.s. planes.
Refinement. Refinement of *F*^2^ against ALL reflections. The weighted *R*-factor *wR* and goodness of fit *S* are based on *F*^2^, conventional *R*-factors *R* are based on *F*, with *F* set to zero for negative *F*^2^. The threshold expression of *F*^2^ > σ(*F*^2^) is used only for calculating *R*-factors(gt) *etc*. and is not relevant to the choice of reflections for refinement. *R*-factors based on *F*^2^ are statistically about twice as large as those based on *F*, and *R*- factors based on ALL data will be even larger.

### Fractional atomic coordinates and isotropic or equivalent isotropic displacement parameters (Å^2^)

**Table:**

	*x*	*y*	*z*	*U*_iso_*/*U*_eq_	
Cl1	1.17644 (10)	−0.26287 (7)	−0.12764 (5)	0.0872 (3)	
S1	0.40720 (6)	0.12989 (5)	0.10363 (3)	0.04261 (19)	
O1	0.9575 (2)	0.11270 (16)	−0.10024 (12)	0.0767 (8)	
H1	0.902 (4)	0.105 (3)	−0.0865 (19)	0.092*	
O2	0.34086 (18)	0.19106 (14)	0.06787 (9)	0.0566 (6)	
O3	0.35672 (17)	0.04443 (14)	0.12555 (9)	0.0533 (5)	
N1	0.82013 (19)	0.03779 (16)	−0.03019 (10)	0.0449 (6)	
N2	0.4449 (2)	0.20143 (17)	0.15497 (11)	0.0484 (6)	
H2	0.442 (3)	0.252 (2)	0.1502 (14)	0.058*	
N3	0.5377 (2)	0.25140 (17)	0.23450 (10)	0.0504 (6)	
N4	0.5464 (2)	0.08644 (17)	0.20508 (10)	0.0494 (6)	
C1	0.9660 (2)	−0.05366 (19)	−0.07463 (12)	0.0438 (6)	
C2	1.0209 (3)	−0.1429 (2)	−0.08168 (13)	0.0535 (8)	
H2A	0.9970	−0.1960	−0.0608	0.064*	
C3	1.1091 (3)	−0.1523 (2)	−0.11879 (14)	0.0546 (8)	
C4	1.1463 (3)	−0.0739 (2)	−0.14968 (16)	0.0685 (10)	
H4A	1.2067	−0.0811	−0.1748	0.082*	
C5	1.0950 (3)	0.0147 (2)	−0.14375 (16)	0.0703 (10)	
H5	1.1202	0.0668	−0.1651	0.084*	
C6	1.0057 (3)	0.0265 (2)	−0.10596 (14)	0.0543 (8)	
C7	0.8701 (2)	−0.0437 (2)	−0.03688 (12)	0.0479 (7)	
H7	0.8442	−0.0973	−0.0171	0.058*	
C8	0.7233 (2)	0.05312 (19)	0.00426 (11)	0.0412 (6)	
C9	0.6811 (3)	0.1460 (2)	0.00458 (14)	0.0565 (8)	
H9	0.7170	0.1935	−0.0168	0.068*	
C10	0.5868 (3)	0.1695 (2)	0.03601 (14)	0.0561 (8)	
H10	0.5597	0.2325	0.0364	0.067*	
C11	0.5325 (2)	0.09899 (18)	0.06706 (11)	0.0390 (6)	
C12	0.5748 (2)	0.00607 (19)	0.06810 (12)	0.0448 (7)	
H12	0.5395	−0.0409	0.0901	0.054*	
C13	0.6696 (2)	−0.01695 (19)	0.03636 (12)	0.0479 (7)	
H13	0.6976	−0.0798	0.0365	0.057*	
C14	0.5142 (2)	0.1773 (2)	0.20062 (11)	0.0432 (6)	
C15	0.6017 (3)	0.2304 (2)	0.27940 (13)	0.0577 (8)	
C16	0.6394 (3)	0.1374 (3)	0.28828 (14)	0.0631 (9)	
H16	0.6841	0.1229	0.3196	0.076*	
C17	0.6107 (3)	0.0666 (2)	0.25061 (14)	0.0552 (8)	
C18	0.6497 (3)	−0.0362 (3)	0.25674 (17)	0.0825 (12)	
H18A	0.6198	−0.0741	0.2262	0.124*	
H18B	0.6227	−0.0618	0.2920	0.124*	
H18C	0.7314	−0.0385	0.2560	0.124*	
C19	0.6268 (4)	0.3114 (3)	0.31925 (16)	0.0894 (13)	
H19A	0.5930	0.3698	0.3053	0.134*	
H19B	0.7078	0.3198	0.3223	0.134*	
H19C	0.5958	0.2966	0.3558	0.134*	

### Atomic displacement parameters (Å^2^)

**Table:**

	*U*^11^	*U*^22^	*U*^33^	*U*^12^	*U*^13^	*U*^23^
Cl1	0.0896 (8)	0.0611 (5)	0.1110 (9)	0.0210 (5)	0.0139 (6)	−0.0059 (5)
S1	0.0421 (4)	0.0436 (4)	0.0421 (4)	0.0046 (3)	−0.0031 (3)	0.0001 (3)
O1	0.0854 (19)	0.0453 (12)	0.099 (2)	0.0069 (12)	0.0452 (16)	0.0174 (12)
O2	0.0549 (13)	0.0587 (12)	0.0562 (13)	0.0154 (10)	−0.0166 (10)	0.0013 (10)
O3	0.0474 (12)	0.0526 (11)	0.0601 (13)	−0.0030 (9)	0.0087 (10)	0.0007 (10)
N1	0.0447 (13)	0.0476 (13)	0.0424 (14)	−0.0036 (11)	0.0031 (11)	−0.0003 (10)
N2	0.0594 (16)	0.0417 (12)	0.0442 (14)	0.0095 (12)	−0.0076 (12)	−0.0026 (11)
N3	0.0539 (15)	0.0534 (14)	0.0440 (14)	−0.0051 (12)	−0.0003 (12)	−0.0005 (11)
N4	0.0525 (15)	0.0515 (14)	0.0442 (14)	0.0134 (11)	−0.0001 (11)	0.0037 (11)
C1	0.0435 (16)	0.0447 (14)	0.0433 (16)	−0.0046 (12)	−0.0021 (13)	−0.0001 (12)
C2	0.060 (2)	0.0440 (15)	0.0562 (19)	−0.0030 (14)	0.0014 (15)	0.0036 (14)
C3	0.0499 (18)	0.0502 (16)	0.064 (2)	0.0036 (14)	0.0001 (15)	−0.0047 (14)
C4	0.056 (2)	0.068 (2)	0.081 (3)	0.0001 (17)	0.0257 (18)	−0.0003 (19)
C5	0.068 (2)	0.0556 (19)	0.087 (3)	−0.0026 (17)	0.030 (2)	0.0141 (18)
C6	0.0564 (19)	0.0460 (16)	0.060 (2)	−0.0028 (14)	0.0102 (16)	0.0026 (14)
C7	0.0500 (17)	0.0491 (15)	0.0447 (16)	−0.0117 (13)	−0.0008 (14)	0.0043 (13)
C8	0.0442 (15)	0.0434 (14)	0.0360 (14)	−0.0049 (12)	0.0003 (12)	−0.0006 (12)
C9	0.063 (2)	0.0436 (16)	0.063 (2)	−0.0043 (14)	0.0172 (16)	0.0136 (14)
C10	0.065 (2)	0.0387 (14)	0.064 (2)	0.0033 (14)	0.0120 (17)	0.0105 (14)
C11	0.0428 (15)	0.0409 (13)	0.0334 (14)	−0.0002 (11)	−0.0030 (11)	0.0017 (11)
C12	0.0522 (17)	0.0375 (14)	0.0446 (16)	−0.0021 (12)	0.0090 (13)	0.0071 (12)
C13	0.0533 (18)	0.0386 (14)	0.0517 (18)	0.0031 (12)	0.0073 (14)	0.0044 (13)
C14	0.0405 (15)	0.0512 (16)	0.0378 (15)	0.0043 (13)	0.0040 (12)	0.0023 (12)
C15	0.0516 (19)	0.076 (2)	0.0454 (18)	−0.0206 (16)	−0.0016 (15)	0.0042 (16)
C16	0.0472 (18)	0.093 (2)	0.0489 (19)	−0.0113 (17)	−0.0124 (15)	0.0199 (18)
C17	0.0459 (17)	0.0693 (19)	0.0504 (18)	0.0065 (15)	0.0032 (14)	0.0173 (16)
C18	0.081 (3)	0.081 (2)	0.086 (3)	0.026 (2)	−0.007 (2)	0.027 (2)
C19	0.105 (3)	0.096 (3)	0.068 (2)	−0.046 (3)	−0.019 (2)	−0.004 (2)

### Geometric parameters (Å, °)

**Table:**

Cl1—C3	1.736 (3)	C5—H5	0.9300
S1—O3	1.422 (2)	C7—H7	0.9300
S1—O2	1.4282 (19)	C8—C9	1.379 (4)
S1—N2	1.629 (3)	C8—C13	1.384 (4)
S1—C11	1.759 (3)	C9—C10	1.371 (4)
O1—C6	1.328 (4)	C9—H9	0.9300
O1—H1	0.74 (4)	C10—C11	1.378 (4)
N1—C7	1.282 (4)	C10—H10	0.9300
N1—C8	1.414 (3)	C11—C12	1.379 (3)
N2—C14	1.393 (4)	C12—C13	1.380 (4)
N2—H2	0.71 (3)	C12—H12	0.9300
N3—C14	1.332 (3)	C13—H13	0.9300
N3—C15	1.333 (4)	C15—C16	1.378 (5)
N4—C14	1.318 (3)	C15—C19	1.495 (5)
N4—C17	1.343 (4)	C16—C17	1.368 (5)
C1—C2	1.404 (4)	C16—H16	0.9300
C1—C6	1.414 (4)	C17—C18	1.503 (4)
C1—C7	1.443 (4)	C18—H18A	0.9600
C2—C3	1.363 (4)	C18—H18B	0.9600
C2—H2A	0.9300	C18—H18C	0.9600
C3—C4	1.380 (4)	C19—H19A	0.9600
C4—C5	1.373 (4)	C19—H19B	0.9600
C4—H4A	0.9300	C19—H19C	0.9600
C5—C6	1.388 (4)		
			
O3—S1—O2	118.89 (13)	C10—C9—H9	119.6
O3—S1—N2	110.33 (13)	C8—C9—H9	119.6
O2—S1—N2	103.22 (12)	C9—C10—C11	119.6 (3)
O3—S1—C11	108.98 (12)	C9—C10—H10	120.2
O2—S1—C11	107.97 (13)	C11—C10—H10	120.2
N2—S1—C11	106.75 (13)	C10—C11—C12	120.3 (3)
C6—O1—H1	107 (3)	C10—C11—S1	118.5 (2)
C7—N1—C8	124.8 (2)	C12—C11—S1	121.3 (2)
C14—N2—S1	126.2 (2)	C11—C12—C13	119.7 (2)
C14—N2—H2	113 (3)	C11—C12—H12	120.1
S1—N2—H2	118 (3)	C13—C12—H12	120.1
C14—N3—C15	115.3 (3)	C12—C13—C8	120.2 (3)
C14—N4—C17	114.9 (3)	C12—C13—H13	119.9
C2—C1—C6	118.5 (3)	C8—C13—H13	119.9
C2—C1—C7	121.1 (3)	N4—C14—N3	128.9 (3)
C6—C1—C7	120.4 (3)	N4—C14—N2	117.3 (3)
C3—C2—C1	120.6 (3)	N3—C14—N2	113.8 (2)
C3—C2—H2A	119.7	N3—C15—C16	120.5 (3)
C1—C2—H2A	119.7	N3—C15—C19	116.7 (3)
C2—C3—C4	120.4 (3)	C16—C15—C19	122.8 (3)
C2—C3—Cl1	120.5 (2)	C17—C16—C15	119.4 (3)
C4—C3—Cl1	119.1 (3)	C17—C16—H16	120.3
C5—C4—C3	120.7 (3)	C15—C16—H16	120.3
C5—C4—H4A	119.7	N4—C17—C16	120.9 (3)
C3—C4—H4A	119.7	N4—C17—C18	116.3 (3)
C4—C5—C6	120.1 (3)	C16—C17—C18	122.8 (3)
C4—C5—H5	119.9	C17—C18—H18A	109.5
C6—C5—H5	119.9	C17—C18—H18B	109.5
O1—C6—C5	119.5 (3)	H18A—C18—H18B	109.5
O1—C6—C1	120.8 (3)	C17—C18—H18C	109.5
C5—C6—C1	119.6 (3)	H18A—C18—H18C	109.5
N1—C7—C1	121.2 (3)	H18B—C18—H18C	109.5
N1—C7—H7	119.4	C15—C19—H19A	109.5
C1—C7—H7	119.4	C15—C19—H19B	109.5
C9—C8—C13	119.2 (3)	H19A—C19—H19B	109.5
C9—C8—N1	115.6 (2)	C15—C19—H19C	109.5
C13—C8—N1	125.2 (2)	H19A—C19—H19C	109.5
C10—C9—C8	120.9 (3)	H19B—C19—H19C	109.5
			
O3—S1—N2—C14	54.0 (3)	O3—S1—C11—C10	171.4 (2)
O2—S1—N2—C14	−178.0 (3)	O2—S1—C11—C10	41.0 (3)
C11—S1—N2—C14	−64.3 (3)	N2—S1—C11—C10	−69.4 (3)
C6—C1—C2—C3	−1.3 (4)	O3—S1—C11—C12	−7.1 (3)
C7—C1—C2—C3	177.9 (3)	O2—S1—C11—C12	−137.5 (2)
C1—C2—C3—C4	0.6 (5)	N2—S1—C11—C12	112.0 (2)
C1—C2—C3—Cl1	−179.7 (2)	C10—C11—C12—C13	−2.2 (4)
C2—C3—C4—C5	−0.3 (6)	S1—C11—C12—C13	176.3 (2)
Cl1—C3—C4—C5	−180.0 (3)	C11—C12—C13—C8	1.0 (4)
C3—C4—C5—C6	0.7 (6)	C9—C8—C13—C12	0.2 (4)
C4—C5—C6—O1	179.4 (4)	N1—C8—C13—C12	−179.4 (3)
C4—C5—C6—C1	−1.5 (6)	C17—N4—C14—N3	0.9 (4)
C2—C1—C6—O1	−179.1 (3)	C17—N4—C14—N2	−178.4 (3)
C7—C1—C6—O1	1.7 (5)	C15—N3—C14—N4	−0.9 (4)
C2—C1—C6—C5	1.8 (5)	C15—N3—C14—N2	178.4 (3)
C7—C1—C6—C5	−177.4 (3)	S1—N2—C14—N4	−4.9 (4)
C8—N1—C7—C1	177.5 (2)	S1—N2—C14—N3	175.7 (2)
C2—C1—C7—N1	179.7 (3)	C14—N3—C15—C16	0.4 (4)
C6—C1—C7—N1	−1.1 (4)	C14—N3—C15—C19	−178.2 (3)
C7—N1—C8—C9	−178.6 (3)	N3—C15—C16—C17	−0.1 (5)
C7—N1—C8—C13	1.1 (4)	C19—C15—C16—C17	178.4 (3)
C13—C8—C9—C10	−0.2 (5)	C14—N4—C17—C16	−0.5 (4)
N1—C8—C9—C10	179.4 (3)	C14—N4—C17—C18	−179.5 (3)
C8—C9—C10—C11	−0.9 (5)	C15—C16—C17—N4	0.2 (5)
C9—C10—C11—C12	2.2 (5)	C15—C16—C17—C18	179.1 (3)
C9—C10—C11—S1	−176.4 (3)		

### Hydrogen-bond geometry (Å, °)

**Table:**

*D*—H···*A*	*D*—H	H···*A*	*D*···*A*	*D*—H···*A*
O1—H1···N1	0.73 (5)	1.90 (4)	2.534 (3)	146 (5)
N2—H2···O1^i^	0.71 (3)	2.22 (3)	2.886 (3)	156 (3)
C9—H9···O2^ii^	0.93	2.48	3.398 (4)	171

Symmetry codes:  (i) *x*−1/2, −*y*+1/2, −*z*; (ii) *x*+1/2, −*y*+1/2, −*z*.
